# Role of gga-miR-221 and gga-miR-222 during Tumour Formation in Chickens Infected by Subgroup J Avian Leukosis Virus

**DOI:** 10.3390/v7122956

**Published:** 2015-12-11

**Authors:** Zhenkai Dai, Jun Ji, Yiming Yan, Wencheng Lin, Hongxin Li, Feng Chen, Yang Liu, Weiguo Chen, Yingzuo Bi, Qingmei Xie

**Affiliations:** 1 College of Animal Science, South China Agricultural University & Guangdong Provincial Key Lab of Agro-Animal Genomics and Molecular Breeding, Guangzhou 510642, China; daizhenkai@stu.scau.edu.cn (Z.D.); wuliaozhiji404@163.com (Y.Y.); Linwencheng@cau.edu.cn (W.L.); lihongxing12@126.com (H.L.); fengch@scau.edu.cn (F.C.); 15813372565@163.coml (Y.L.); Wgchen81@scau.edu.cn (W.C.); yizbi@126.com (Y.B.); 2 China-UK-NYNU-RRes Joint laboratory of Insect Biology, Nanyang Normal Universiy, Nanyang 473000, China; jijun84@gmail.com; 3 Institute of Animal Science, Guangdong Academy of Agriculture Sciences, Guangzhou 510640, China; 4 Key Laboratory of Animal Health Aquaculture and Environmental Control, Guangzhou 510642, China; 5 South China Collaborative Innovation Center for Poultry Disease Control and Product Safety, Guangzhou 510640, China

**Keywords:** subgroup J avian leukosis virus, gga-miR-221, gga-miR-222, BMF, apoptosis, cancer, cisplatin, doxorubicin

## Abstract

Subgroup J avian leukosis virus (ALV-J) causes a neoplastic disease in infected chickens. Differential expression patterns of microRNAs (miRNAs) are closely related to the formation and growth of tumors. (1) Background: This study was undertaken to understand how miRNAs might be related to tumor growth during ALV-J infection. We chose to characterize the effects of miR-221 and miR-222 on cell proliferation, migration, and apoptosis based on previous microarray data. (2) Methods: *In vivo*, the expression levels of miR-221 and miR-222 were significantly increased in the liver of ALV-J infected chickens (*p* < 0.01). Over-expression of gga-miR-221 and gga-miR-222 promoted the proliferation, migration, and growth of DF-1 cells, and decreased the expression of BCL-2 modifying factor (BMF) making cells more resistant to apoptosis. (3) Results: Our results suggest that gga-miR-221 and gga-miR-222 may be tumour formation relevant gene in chicken that promote proliferation, migration, and growth of cancer cells, and inhibit apoptosis. BMF expression was significantly reduced *in vivo* 70 days after ALV-J infection. They may also play a pivotal role in tumorigenesis during ALV-J infection.

## 1. Introduction

The subgroup J Avian Leukosis virus (ALV-J) can form tumors on chickens and can thus lead to their death; it also can cause non-tumor diseases, namely the secondary infection caused by immunosuppression [1]. The symptoms are emaciation, anemia, tolerance viremia, and immunosuppression, all of which are able to seriously affect poultry production [2,3]. ALV-J has affected the poultry industry worldwide, with the exception of New Zealand. It has been detected in America [4], the Middle East [5], Australia [6], and has caused substantial economic losses.

Tumors are a kind of disease of poly-genetic abnormality and regulatory disorder. The forming mechanism of it is that carcinogenic factor activates the division of normal cells, breaks the limit of the cell cycle [7] and inhibits apoptosis [8]. This will gradually turn these normal cells into cancer cells which are able to automatically and abnormally proliferate with astonishing speed, and transfer to and invade other organs through the circulation system. Apoptosis is a conserved form of cell death that plays a pivotal role in the formation of cells and the stability of tissues. If apoptosis becomes dysfunctional, it can lead to cancer and autoimmunity. Apoptosis can be triggered through several different pathways. Apoptosis is regulated in part by the BCL-2 family of proteins. The BCL-2 family contains both apoptosis-inhibitors and apoptosis-inducers that act to maintain the stability, structure, and function of mitochondria [9,10,11,12]. BCL-2 modifying factor (BMF) is an apoptosis—inducing member of the BCL-2 family in the bcl-2 homology domain only proteins (BH3-only) sub-family, named for having only one characteristic BH3 protein domain [13]. The BH3-only proteins likely activate other apoptosis effectors, like Bax and Bak. Activation may occur directly, for example, Bim, Bid [14,15], and p53-upregulated mediator of apoptosis (PUMA) [16] bind to Bax forming oligomers that induce apoptosis; or indirectly, for example, PUMA [17], Bad, and BMF [18] bind to apoptosis-inhibitors (BCL-2 or BCL-x1) and replace them with apoptosis-inducers (Bax and Bak). Chicken bmf can induce apoptosis after transfection in Michigan Cancer Foundation-7(MCF7) and HeLa cell lines [19]. In a mouse model, absence of a single Bim allele exacerbates leukemia [20], and Bim deficiency can accelerate the development of cancer by allowing cells to survive [21]. Thus, the BH3-only proteins are recognized as tumor inhibitors.

MicroRNA (miRNA, miR) is a non-coding single-stranded RNA molecule approximately [22] base pairs in length that regulates gene expression [22,23]. Calin *et al.* [24] were the first to provide evidence of the essential role of miRNA disorders in the process of tumor-formation. MiR-15 and miR-16 are located in the 13q14 chromosome region, the partial absence of which was strongly influential in an outbreak of chronic lymphocytic leukemia (CLL). In addition, miR-15 and miR-16 are negatively correlated with expression of the anti-apoptotic gene BCL-2 [25], which inhibits apoptosis at the level of the mitochondria and is critical for cancer cells [26]. In poultry, Li *et al.* [27], have reported gga-miR-375 may function as a tumor suppressor in infection of ALV-J.

MiR-221 and miR-222, are examples of miRNA that is highly conserved among chordates and is located on the X chromosome in humans, rats, and mice [28]. In chickens, miR-221 and miR-222 are located on chromosome 1. Our previous study has shown that gga-miR-221 and gga-miR-222 to be frequently up-regulated in the livers of chickens 10 weeks post ALV-J infection [29]. Increased expression of these miRNAs has been extensively documented in many different types of tumors including nervous system leukemia [30,31], breast cancer [32], prostate cancer [33] and gastric cancer [34]. Given the over-expression levels of miR-221 and miR-222 in terminal malignant tumors, they are likely therapeutic targets for carcinoma. Galardi *et al.* [35], suggested that the regulatory factor of cell cycle p27 (Kip l) may be a target of miR-221 and miR-222 regulation, and found that the quantity of p27 in pancreas cells was negatively correlated with the expression level of miR-221 and miR-222, suggesting they likely regulate cell proliferation and the cell cycle. Inhibition of miR-221 and miR-222 could possibly inhibit growth and induce apoptosis in cells [36]. Gramantieri *et al.* [37], demonstrated that BMF is a target of miR-221 and may play a role in mediating miR-221-induced inhibition of apoptosis in hepatocellular carcinoma. Lambeth *et al.* [38], have reported increased expression of *Gallus gallus* (gga)-miR-221 and gga-miR-222 in poultry in the cancer cell line MSB-1 transformed by MDV (Marek’s Disease Virus), suggesting a link between oncogenic poultry viruses and expression levels of miR-221 and miR-222. They also provided evidence that gga-miR-221 and gga-miR-222 inhibit p27 (Kip l). Despite the link to increased expression of miR-221 and miR-222 in MDV infection, there has not been a comprehensive study assessing gga-miR-221 and gga-miR-222 expression in chickens infected by ALV-J.

To understand more fully whether there is a correlation between differential expression of gga-miR-221 and gga-miR-222 and ALV-J pathogenesis, we examined the functions of gga-miR-221 and gga-miR-222 in cell proliferation, migration, and apoptosis *in vitro*, and determined the expression levels of gga-miR-221 and gga-miR-222 in ALV-J infection *in vivo*.

## 2. Materials and Methods

### 2.1. Virus and Cell Lines

The NX0101 strain of ALV-J was graciously provided by Cui Zhizhong from Shangdong Agricultural University, People’s Republic of China. It was isolated from a parent breeder farm of white meat-type in 2001 [39]. The DF-1 was an immortalized chicken embryo fibroblast cell line, and was obtained from American Type Culture Collection (Manassas, VA, USA). The DF-1 cells were stored in Dulbecco’s modified eagle medium (DMEM) supplemented with 10% fetal bovine serum (FBS).

### 2.2. Cell Transfections

DF-1 cells were transfected with miRNA mimics for gga-miR-221, gga-miR-222 (Shanghai GenePharma Co., Ltd., Shanghai, China) or Negative control mimics (named gga-miR-NC; designed as described previously) using X-tremeGENE siRNA Transfection Reagent (Roche Applied Science, Mannheim, Germany) as the transfection reagent. For each transfection, 40 nM of miRNA mimics were respectively used in a six-well plate, unless otherwise indicated. Non-transfected cells were used as the blank control (mock group).

### 2.3. Cell Proliferation Assay

DF-1 cells were grown in DMEM supplemented with 10% FBS in 96-well plates at 1.0 × 10^4^ cells/well. Twenty-four hours after the inoculation, the DF-1 cells were divided into four equal groups. MiRNA mimics for gga-miR-221, gga-miR-222, and the NC mimics were transfected into DF-1 cells in triplicate at 40 nM. The fourth group of mock transfected DF-1 cells were established in parallel as a control. The DF-1 cells were stained with 10 μL of WST-1 (cell proliferation dye) 24, 48, and 72 h after transfection, incubated for 2 h at 37 °C, and then the absorbance was read at 450nm using a multimode microplate reader.

### 2.4. Colony Formation Assay

DF-1 cells were grown in 24-well plates at 1.6 × 10^5^ cells/well. Twenty-four hours after plating, gga-miR-221, gga-miR-222, and negative control mimics were transfected into the DF-1 cells in triplicate. A fourth mock transfected group was established in parallel as a control. To separate the cells, pancreatin was added 24 h after the transfection. The DF-1 cells were seeded in six-well plates at 1000 cells/well, and grown in DMEM + 10% FBS for two weeks. The DMEM was replaced every three days. At the end of two weeks, the cells were washed with PBS and immobilized on the plates for 15 min through using methyl alcohol. After the methyl alcohol was removed, the cells were stained with 0.1% crystal violet for 15 min, and then washed three times with PBS. The plates were photographed to determine the number of colonies.

### 2.5. Wound Healing Assay

Gga-miR-221, gga-miR-222, and normal control (NC) mimics transfected into DF-1 cells in triplicate in 24-well plates at 1.6 × 10^5^ cells/well. A mock transfected group was established in parallel as a control. Twenty-four hours after the transfection, a 200 uL yellow pipette tip was used to scratch the cells growing on the plates, even pressure was applied throughout, to create a “wound” *in vitro*. Unattached cells were removed by washing twice with complete medium. The cells were photographed under a microscope at 0, 24, and 48 h post wounding.

### 2.6. Analysis of Cell Apoptosis

The cell apoptosis index was determined using AnnexinV-FITC/Propidium iodide (PI) staining and DAPI to identify nuclei. DF-1 cells were transfected with the gga-miR-221, gga-miR-222, and NC mimics in six-well plates at 2 × 10^5^ cells/well. A mock transfected group was established in parallel as a control. Twenty-four hours after transfection, the cells were serum starved for 48 h, treated with cisplatin (0.5 μg/mL) for 72 h, or treated with doxorubicin (0.2 μg/mL) for 72 h. Annexin V-FITC/PI apoptosis assay was conducted using the Annexin V-FITC/PI apoptosis assay kit (Beyotime Insititute of Biotechnology, Shanghai, China) according to the manufacturer’s instructions. Cell morphology was observed utilizing fluorescence microscope and a nuclear DAPI (Beyotime Insititute of Biotechnology) stain. The rate of apoptosis was determined using the following formula: apoptosis rate (%) = number of apoptotic cells/ total cell number (the total cell number is equal to or greater than 500).

### 2.7. Vector Construction

To construct luciferase reporter vectors, pmiRGLO-BMF-3′UTR-wt and pmiRGLO-BMF-3′UTR-mut, wild-type 3′UTR fragment of BMF were amplified by RT-PCR using the primers 5′-GTTCTCGAGGGAGCACTGAACAAACACT-3′ and 5′-GTGGTCTAGACACGAAGCAAATATACTGCACA-3′.mutant-type of BMF were amplified by RT-PCR using the primers 5’-AACCTGCATG TCTCACTGGGTCTCC-3′ and 5′-GAGAGGACGTACTTTTGTTTATCCTGGTT-3′. The amplified putative binding sites for miR221 and miR-222 in the 3′ UTR sequence were inserted downstream of the stop codon of firefly luciferase in the pmiR-GLO Dual-Luciferase miRNA Target Expression Vector (Ambion, Promega, Beijing, China). The 3′UTR-pmiR-GLO recombinant plasmids were identified by PCR and restriction enzyme analysis, and then selected for further sequencing.

### 2.8. Luciferase Activity Assay

DF-1 cells were grown in 24-well plate at 3.4 × 10^5^ cells/well. The cells reached 80%–90% confluence after 24 h. The DF-1 cells were transfected 10 nmol/L gga-miR-221 and gga-miR-222 mimics 20 ng BMF-3’UTR-wt, or BMF-3’UTR-mut, and 4 ng pRL-TK (Promega). Luciferase activity was determined 48 h after the transfection using the Dual-Glo^®^ Luciferase Assay System kit according to the manufacturer’s instructions.

### 2.9. Western Blot

Gga-miR-221, gga-miR-222, and NC mimics were transfected into DF-1 cells in triplicate in 24-well plates at 1.6 × 10^5^ cells/well. A mock transfected group was established in parallel as a control. The cells were lysed and the protein extracted 48 h after the transfection. BMF was detected using a Rabbit anti-Human BMF polyclonal antibody (1:1000; Abcam, Cambridge, UK) and a goat anti-rabbit IgG-HRP (1:5000; Proteintech Group, Inc., Chicago, IL, USA). The loading control was β-actin (1:600; predicted molecular weight: 42 kDa; Proteintech Group, Inc.).

### 2.10. Animal Infection Assay

One-day-old SPF pullets (*n* = 200) were randomly divided into a control group and an infection group. The pullets in the control group were inoculated with an equivalent volume of sterilized normal saline in the enterocoelia, and the pullets in the infected group received ALV-J NX0101 at 10^3.7^ tissu culture infective dose TCID_50_ in 0.2 mL. Both of the groups were fed in a negative pressure isolator, and were offered food and water *Ad libitum*. During the course of the experimental infection, 100 chickens were infected with no deaths reported. All animals were euthanized and autopsied upon completion of the experiments. At the early stage of infection, chickens in the infection group were as healthy as those in the control group. After 40 day post infection, chickens in the infected group presented symptoms of anemia, such as inappetence, pale comb, cooling-down of chicken feet as well as progressive emaciation. Compared to the chickens in control group, Chickens in the infected group had histopathological changes, such as hepatomegaly and splenomegaly. Beginning at 10-days old, the pullets (*n* = 6) were weighed every 10 days and sacrificed for testing. Morbidity was observed and documented. Tissue samples were obtained from the liver, spleen, marrow, and blood and stored at −70 °C until miRNA and mRNA extraction. The experiment lasted 90 days and nine samples were obtained. Use of animals in this study was approved by the South China Agricultural University Committee of Animal Experiments (approval ID 201004152) (Guangzhou, China).

### 2.11. Real-Time Quantitative RT-PCR

Total RNA was extracted from the liver, spleen, and blood samples taken from the pullets in the control and infected groups at each time point using TRIZOL (Invitrogen, Carlsbad, CA, USA). The miRNA was extracted using Isolation of small and large RNA (Macherey-Nagel, Berlin, Germany).

The relative expression levels of gga-miR221, gga-miR-222, bmf by RT-qPCR. Gga-miR221 and gga-miR-222 expression was normalized to the 5s snRNA (chicken), and Bmf were normalized to HMBS (chicken). gga-miR221 and gga-miR-222 were amplified by RT-qPCR using Exiqon company design the miRNA primers.Bmf were amplified by RT-qPCR using the primers5’–3’TCCAAGAAGAGCCTCAGGAA and 5’–3’AAGAGAAAAAGCTGCCACCA The sequences of interest were transcribed into cDNA for the RT-qPCR reaction using the miRCURY LNA™ Universal RT microRNA PCR, SYBR Green master mix (Exiqon, Copenhagen, Denmark). The relative expression levels of gga-miR-221, gga-miR-222, and Bmf were calculated using the 2^−△△^*^C^*^T^ method.

### 2.12. Data Analysis

Data analysis was conducted using SPSS version 19.0 (IBM, Armonk, NY, USA). The homogeneity of variance was tested using the Levene’s test of homogeneity of variance. If the variance was homogenous, Least-significant difference (LSD) inspection was carried out. If the variance was not homogenous, Dunnett’s T3 inspection was performed. The Pearson linear correlation analysis was performed, and a p value less than 0.05 was established as the criterion for significance.

### 2.13. Ethics Statement

The above experiments were conducted in accordance with the institutional and national guidelines for the use and care of laboratory animals. The approval of using animals during the process of this study was obtained from South China Agricultural University Committee for Animal Experiments (approved ID: 201004152).

### 2.14. Accession Number

The microarray data were MIAME compliant which have been deposited in a MIAME compliant database (ArrayExpress, GEO ID: GSE28434). We have deposited the sequences of gga-miR-221 and gga-miR-222 (MI0001178, MI0001177) described in this paper in miRBase.

## 3. Results

### 3.1. Expression of gga-miR-221 and gga-miR-222 in the Liver and Marrow of Pullets Infected with ALV-J

**Figure 1:**
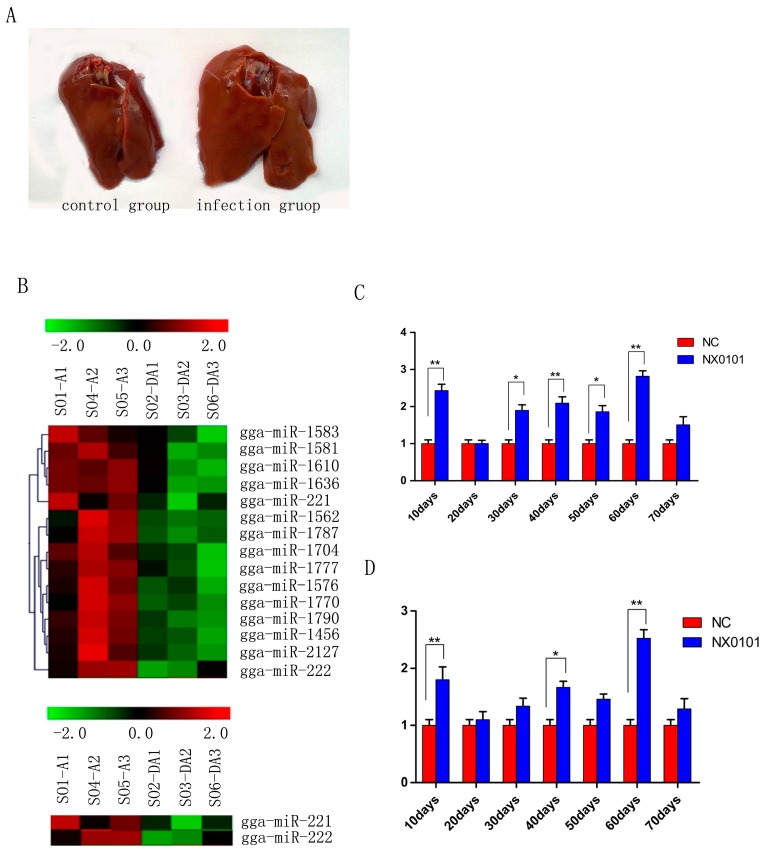
gga-miR-221 and gga-miR-222 expression was upregulated in ALV-J induced cancer. (**A**) Liver lesions in SPF white leghorn chickens induced by viral infection at 70 days; (**B**) Microarray analysis of miRNA expression during ALV-J infection. DA represents the control group and A represents tte infection group; (**C**) and (**D**) gga-miR-221 and gga-miR-222 expression levels in the liver of ALV-J infected chickens were quantified by qRT-PCR every 10 days from 10 to 70 days (** *p* < 0.01; * *p* < 0.05).

Most of chickens in the ALV-J infected group gradually appeared in a comparatively emaciated and weak way by contrast with those of chickens in the control group. At 10 weeks, the size of livers of the infected chickens was obviously greater than that of the control group (Figure 1A). We first examined the expression levels of gga-miR-221 and gga-miR-222 *in vivo* in pullets infected with ALV-J. Gene chip analysis indicated that miR-221 and miR-222 were up-regulated significantly in the livers of infected pullets 10 days post infection (Figure 1B). QRT-PCR was used to confirm that the levels of gga-miR-221 were significantly up-regulated in the livers of the infected pullets compared to the control group at each time point examined, except for the 20-day-old time point (*p* < 0.01; Figure 1C). Likewise, qRT-PCR confirmed that gga-miR-222 levels were significantly increased in the infected group compared to the control group at each time point (*p* < 0.01; Figure 1D).

### 3.2. gga-miR-221 and gga-miR-222 Accelerate the Proliferation, Growth, and Migration of DF-1 Cells

**Figure 2:**
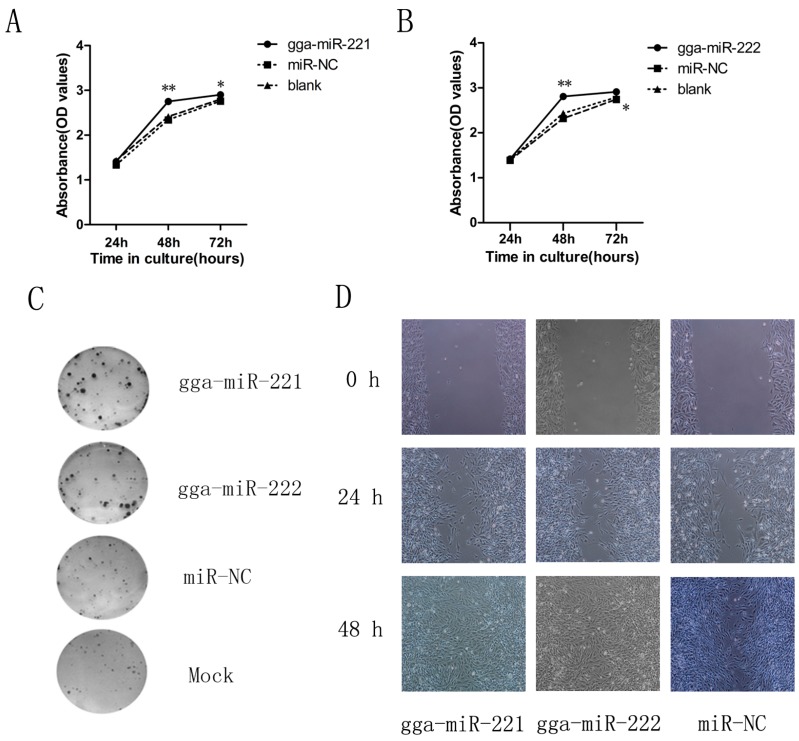
gga-miR-221 and gga-miR-222 promoted DF-1 cell proliferation and invasion. Cells were transfected with gga-miR-221, gga-miR-222, miR-NC, or mock transfected and then underwent WST-1 analysis, wound healing assay, and colony formation assay. (**A**) and (**B**) The effects of gga-miR-221 and gga-miR-222 on proliferation were detected using WST-1 analysis over 48 h. The figures show the means and standard errors from three independent experiments; * *P* value (*p)* ** *p* < 0.01. (**C**) The effects of gga-miR-221 and gga-miR-222 on colony formation of DF-1 cells is shown. (**D**) 48 h after transfection, scratch wounds were made on confluent monolayer cultures. Images of the wound repair process were taken at 0, 24, and 48 h after wounding. Magnification is 200×.

To understand the possible impacts of gga-miR-221 and gga-miR-222 on ALV-J tumorigenesis, we assessed the effects of over-expressing the miRNAs on the proliferation of DF-1 cells over three days. Both gga-miR-221 and gga-miR-222 accelerated the proliferation of DF-1 cells compared to the NC group and the mock transfected cells (Figure 2A,B). There was no apparent difference in proliferation 24 h after transfection between the groups, but at 48 h after the transfection, the cells transfected with gga-miR-221 and gga-miR-222 had the highest levels of proliferation, which was confirmed by the colony formation assay (Figure 2C). The impacts of gga-miR-221 and gga-miR-222 on the growth and migration of DF-1 cells were assessed using an *in vitro* wound healing assay (Figure 2D). Differences in wound healing were apparent 24 h after the transfection, between the experimental groups, the NC group, and the mock transfected group. By 48 h, there is a clear disparity between the experimental groups in which all the cells have completed the healing process and the other groups where there remains a visible growth gap between cells. These results provided additional support that gga-miR-221 and gga-miR-222 effect the growth and migration of DF-1 cells.

### 3.3. gga-miR-221 and gga-miR-222 Inhibit Apoptosis

**Figure 3:**
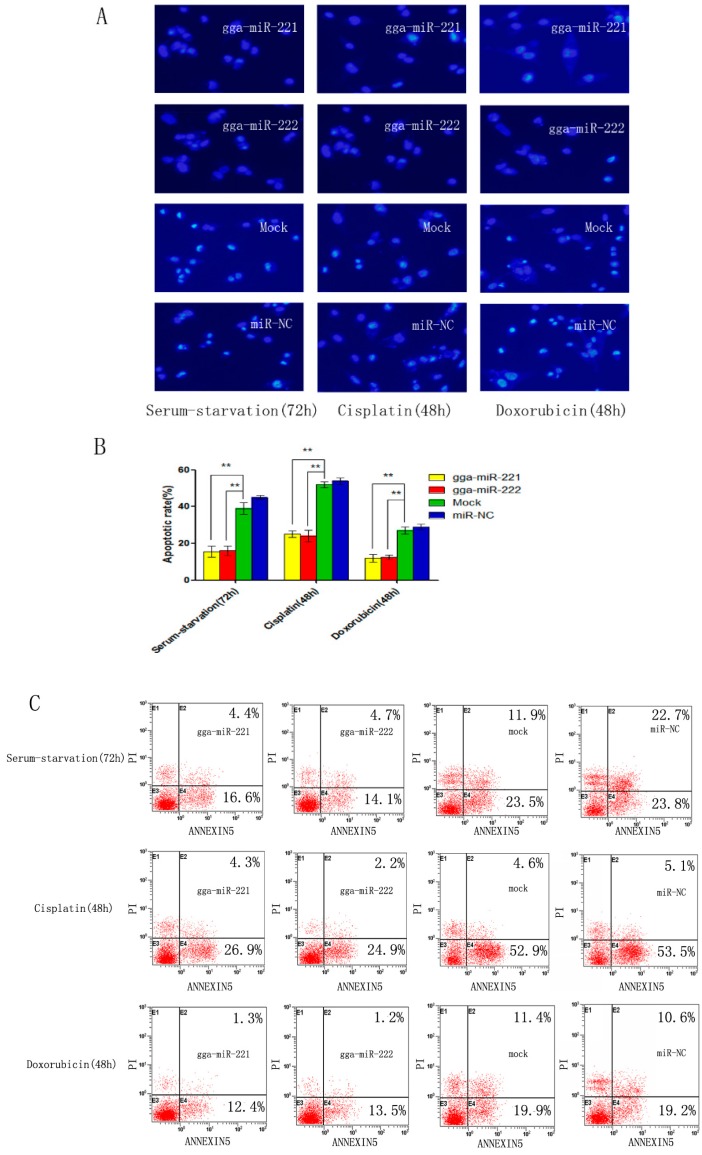
gga-miR-221 and gga-miR-222 inhibit apoptosis induced by serum starvation, cisplatin, and doxorubicin. Cells were transfected with gga-miR-221, gga-miR-222, miR-NC, or mock transfected and then stained with DAPI. (**A**) The rates of apoptosis were evaluated by assessing the apoptotic morphology of the cells. Magnification is 200× (**B**) The differences in the rate of apoptosis between gga-miR-221, gga-miR-222, miR-NC, and mock transfected cells are shown. The graphs show the means and standard errors from three independent experiments ** *p* < 0.01. (**C**) The cells transfected with gga-miR-221, gga-miR-222, miR-NC, or mock transfected and then stained with Annexin V-FITC/PI 48 or 72 h post-transfection.

DF-1 cells were transfected with gga-miR-221, gga-miR-222, NC miRNA, or mock transfected and then induced to undergo apoptosis using serum starvation (48 h), cisplatin treatment (72 h), or doxorubicin treatment (72 h). The level of apoptosis level in DF-1 cells that were transfected with gga-miR-221 and gga-miR-222 was substantially reduced compared to the NC groups and the control group, suggesting that the over-expression of gga-miR-221 and gga-miR-222 has anti-apoptotic effects (Figure 3A,B). The percentage of cells undergoing apoptosis and the stage of apoptosis were assessed by flow cytometry using Annexin V-FITC/PI staining. The percentage of cells undergoing apoptosis after transfection with gga-miR-221 was: 21% after serum starvation, 31.1% after cisplatin treatment, and 13.7% after doxorubicin treatment. Similarly, the percentage of cells transfected with gga-miR-222 undergoing apoptosis were: 18.8% after serum starvation, 25.1% after cisplatin treatment, and 14.8% after doxorubicin (Figure 3C). These results reaffirmed the anti-apoptotic effects of over-expressing gga-miR-221 and gga-miR-222.

### 3.4. gga-miR-221 and gga-miR-222 Inhibit the Expression of BMF by Binding to the 3′-Untranslated Region (UTR)

**Figure 4:**
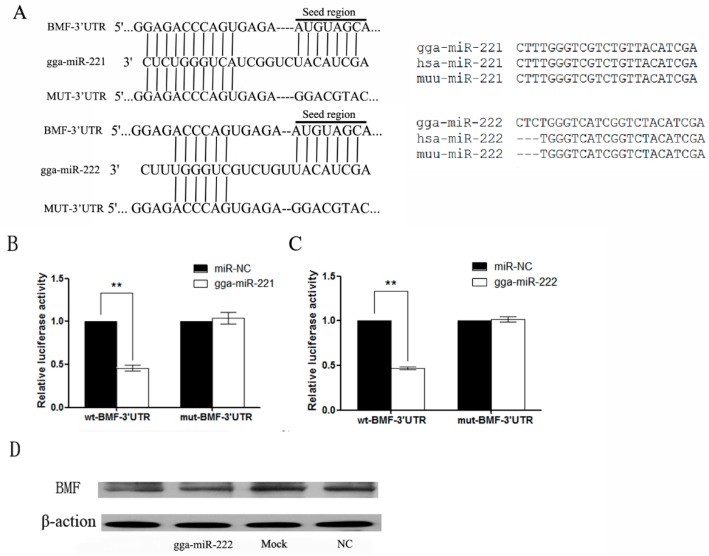
BMF is a direct target for gga-miR-221 and gga-miR-222. (**A**) Alignment of BMF-3’UTR, gga-miR-221,gga-miR-222 and MUT-3’UTR, where the complementary site for the seed region of gga-miR-221 and gga-miR-222 are indicated; (**B**) Differences in gga-miR-221/222, *homo sapiens* miR-221/222, and *Mus musculus* miR-221/222; (**C**) and (**D**) The regulation of luciferase activity from the BMF-3′ UTR is dependent on gga-miR-221 and gga-miR-222. DF-1 cells were co-transfected with gga-miR-221, gga-miR-222, miR-NC and either wt-BMF-3′ UTR (left) or mut-BMF-3′ UTR (right). The graphs show the means and standard errors of at least three independent experiments. ** *p* < 0.01, compared to miR-NC-transfected cells; (**E**) Ectopic expression of gga-miR-221 and gga-miR-222 reduced the level of BMF protein expression in DF-1 cells. β-action levels were used as a control. Each experiment was repeated three times, and each sample was assayed in triplicate.

The target genes of gga-miR-221 and gga-miR-222 were predicted utilizing online databases such as TargetScan, PicTar, and MicroCosm. RNAhybrid was used to increase the accuracy of the results through a functional analysis. The predicted results identified BMF as a target gene of gga-miR-221 and gga-miR-222 (Figure 4A). The gga-miR-221 is identical to *Homo sapiens* (hsa)-miR-221 and *Mus musculus* (muu)-miR-221 while the gga-miR-222 differs from hsa-miR-222 and muu-miR-222 by three bases (Figure 4B). The 3′ UTR sequence containing the miRNA binding sites was cloned into the reporter vector pmiR-GLO (BMF-3′ UTR-wt). A mutant form of the BMF-3′ UTR was also established (BMF-3′ UTR-mut). The wild-type and mutant reporter vectors, and the miRNA and NC mimics transfected into DF-1 cells. The miRNA constructs inhibited BMF-3′ UTR-wt by approximately 50%, but had no effect on expression on the BMF-3′ UTR-mut construct (*p* < 0.01; Figure 4C). DF-1 cells were transfected with gga-miR-221 and gga-miR-222 for 72 h and then the proteins were harvested for Western blot. The level of BMF protein in cells over-expressing gga-miR-221 and gga-miR-222 were reduced compared to the NC and control groups (Figure 4D), suggesting that over-expressed gga-miR-221 and gga-miR-222 inhibit the expression of BMF.

### 3.5. The Expression of mRNA of BMF on Liver, Blood, and Spleen

**Figure 5:**
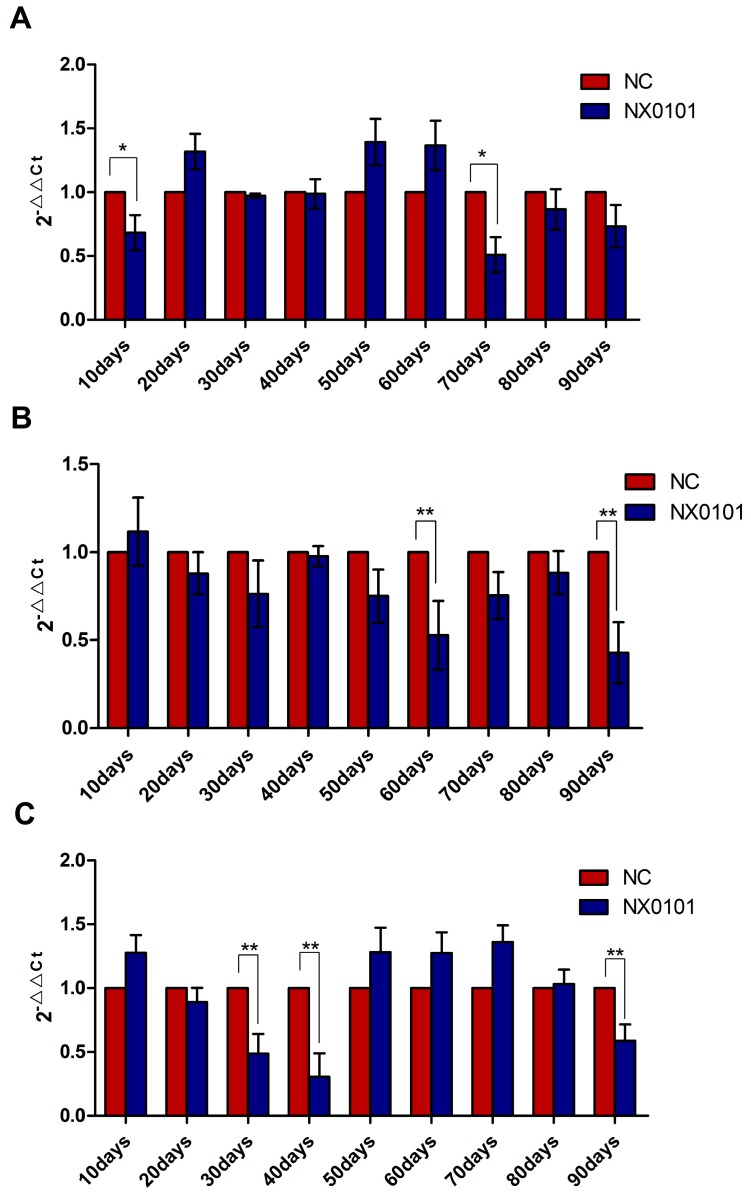
mRNA expression of BMF in the liver, spleen, and blood of chickens infected by ALV-J. (**A**) BMF mRNA expression levels in the liver of infected pullets from 10 to 90 days post infection. (**B**) BMF mRNA expression levels in the spleen of infected pullets from 10 to 90 days post infection. (**C**) BMF mRNA expression levels in the blood of infected pullets from 10 to 90 days post infection. ** *p* < 0.01, * *p* < 0.05.

Given that gga-miR-221 and gga-miR-222 promote the proliferation, migration, and growth of DF-1 cells, and inhibit the apoptosis of DF-1 cells by suppressing the expression of BMF, we assessed the expression level of BMF mRNA in SPF chickens infected with ALV-J. In the liver, the level of BMF mRNA was downregulated 10 and 70 days after infection (Figure 5A). In the spleen, the level of BMF mRNA was downregulated 60 and 90 days after infection (*p* < 0.05; Figure 5B). Finally, in the blood the level of BMF mRNA expression was downregulated 30, 40, and 90 days after infection (*p* < 0.05; Figure 5C).

## 4. Discussion

In this study, we provide evidence that gga-miR-221 and gga-miR-222 are up-regulated in the livers of pullets infected with ALV-J. Furthermore, we demonstrate that gga-miR-221 and gga-miR-222 have stimulating effects on cell growth and proliferation, and that BMF is a likely target for gga-miR-221 and gga-miR-222. Taken together, our results and studies in the Marek’s disease virus (MDV) model suggest that up-regulation of gga-miR-221 and gga-miR-222 might be a general feature of oncogenic poultry viruses.

To better understand the anti-apoptotic function of gga-miR-221 and gga-miR-222, apoptosis was induced using three different methods, serum starvation, cisplatin treatment, and doxorubicin treatment. It is well established that tumor cell proliferation exceeds the limitations of normal cells, and because of their rapid growth, they are constantly challenged by nutrient deficiency. Thus, understanding the anti-apoptotic capabilities of tumor cells under conditions of malnutrition is critical. Serum starved DF-1 cells that were transfected with gga-miR-221 and gga-miR-222 displayed relatively low rates of apoptosis, confirming that gga-miR-221 and gga-miR-222 are anti-apoptotic when cells are malnourished. Cisplatin, a broad-spectrum chemotherapeutic, remains in widespread clinical use. It inhibits tumor cell DNA replication and destroys the structure of the cell membrane resulting in apoptosis. In our study, cells transfected with gga-miR-221 and gga-miR-222 were relatively resistant to cisplatin-induced apoptosis. Similarly, cells transfected with gga-miR-221 and gga-miR-222 were resistant to doxorubicin-induced apoptosis.

Over-expression of miR-221 is associated with a more aggressive phenotype of hepatocellular carcinoma and inhibits apoptosis by binding to BMF. In addition to its own pro-apoptotic function, the BMF gene can regulate the pro-apoptotic function of BCL-2 by binding to it [40]. As it has been confirmed as the target gene of gga-miR-221/222 in our experiments. The qRT-PCR data showed that BMF was down-regulated in the spleen at each time point assessed, and was downregulated in the liver and blood during the later period of infection, which may contribute to ALV-J developing into a chronic disease.

MDV is well studied by scholars in the field as it has caused many cases of acute tumor diseases. Unlike ALV-J, MDV contains a cancer gene in its genome and miRNAs that promote the development of cancer. MDV causes several types of cancer in different organs; and differential expression of miRNAs during MDV infection have been reported [41,42]. Distinct from MDV and other viruses that contain a proto-oncogene, The non-oncogene-bearing retrovirus causes tumors by inserting into the host gene resulting in an insertional mutation [43]. During the lasting period of incubation, cumulative insertional mutations stimulate traditional proto-oncogenes and triggers miRNA dysfunction [44]. Therefore, analyses to detect the expression of miRNAs and genes could possibly elucidate the tumorigenic mechanisms of ALV-J. Taken together, our results suggest that miR-221 and miR-222 regulate BMF and inhibit apoptosis through the mitochondrial pathway. Thus, by regulating miR-221 and miR-222, ALV-J, promotes the proliferation, migration, and growth of cells, and inhibits apoptosis to induce tumor formation.
